# Motion synchronisation patterns of the carotid atheromatous plaque from B-mode ultrasound

**DOI:** 10.1038/s41598-020-65340-2

**Published:** 2020-07-08

**Authors:** Spyretta Golemati, Eleni Patelaki, Aimilia Gastounioti, Ioannis Andreadis, Christos D. Liapis, Konstantina S. Nikita

**Affiliations:** 1 Biomedical Simulations and Imaging Lab., School of Electrical and Computer Engineering, National Technical University of Athens, Athens, Greece; 2 Medical School, National and Kapodistrian University of Athens, Athens, Greece; 3 Institute of Communication and Computer Systems, Athens, Greece; 4 Department of Radiology, University of Pennsylvania, Philadelphia, USA; 5 Attikon University General Hospital, Medical School, National and Kapodistrian University of Athens, Athens, Greece

**Keywords:** Ultrasonography, Biomedical engineering

## Abstract

Asynchronous movement of the carotid atheromatous plaque from B-mode ultrasound has been previously reported, and associated with higher risk of stroke, but not quantitatively estimated. Based on the hypothesis that asynchronous plaque motion is associated with vulnerable plaque, in this study, synchronisation patterns of different tissue areas were estimated using cross-correlations of displacement waveforms. In 135 plaques (77 subjects), plaque radial deformation was synchronised by approximately 50% with the arterial diameter, and the mean phase shift was 0.4 s. Within the plaque, the mean phase shifts between the displacements of the top and bottom surfaces were 0.2 s and 0.3 s, in the radial and longitudinal directions, respectively, and the synchronisation about 80% in both directions. Classification of phase-shift-based features using Random Forests yielded Area-Under-the-Curve scores of 0.81, 0.79, 0.89 and 0.90 for echogenicity, symptomaticity, stenosis degree and plaque risk, respectively. Statistical analysis showed that echolucent, high-stenosis and high-risk plaques exhibited higher phase shifts between the radial displacements of their top and bottom surfaces. These findings are useful in the study of plaque kinematics.

## Introduction

The carotid atheromatous plaque is a lesion of the carotid artery wall and typically consists of a fibrous cap (mostly smooth muscle cells, collagen and elastic fibers) of varying thickness and a lipid core (mostly cholesterol and cellular debris). In cases of advanced degeneration, plaque lesions present a more complicated structure, including calcification, intraplaque hemorrhage and ulceration^1^ and narrow the arterial lumen, obstructing blood flow and oxygen supply to the brain. More severe damage may be caused by vulnerable plaques, i.e. plaques prone to rupture. These are strongly associated with the formation of blood clots and the release of plaque fragments into the systemic circulation, which may cause a cerebrovascular event, such as stroke or transient ischemic attack (TIA)^2^. Given the substantial burden of stroke (15 million people worldwide suffer a stroke annually, of whom 5 million die and 5 million are left permanently disabled^3^), investigating the behaviour of carotid plaque towards improving stroke prevention is of utmost importance.

Ultrasound imaging is the preferred imaging modality for the diagnosis of carotid atheromatous plaque, owing to a number of advantages, including noninvasiveness, bedside availability, short examination times, lack of radiation exposure, and low cost^4^. Currently, clinical management of carotid plaque is based on the degree of stenosis, i.e., the percentage of lumen area occupied by atheromatous material, and the prior occurrence of symptoms^5^. Although the degree of stenosis is a validated marker for management of carotid plaques, some studies have indicated that a high degree of stenosis is not necessarily related to a high risk of a cerebrovascular event^6,7^. These facts indicate that there is room for improving the current clinical scheme for assessing plaque vulnerability, possibly through the identification of noninvasive, low-cost and reliable imaging markers for predicting strokes^8^.

For instance, carotid motion analysis estimated with ultrasound image sequences has gained increasing attention as a potential index of plaque vulnerability^9–12^. Motion analysis can be defined as the estimation of arterial tissue displacement during one or more cardiac cycles. It has been shown that carotid atheromatous plaque performs a complex, multidirectional, often periodic, motion during the cardiac cycle^13^. Despite the technical challenges, such as the low image resolution in ultrasound imaging and the complexity of the local tissue geometry and mechanics, several studies have suggested a number of kinematic and strain indices associated with plaque rupture risk^14^.

A number of efforts have focused on motion of non-atheromatous segments of the arterial wall in normal^15,16^ and pathological conditions, such as hypertension, diabetes and coronary artery disease^17,18^, as well as the motion of the wall adjacent to carotid plaque^12,16,19,20^. These studies have studied the expected cyclical motion in the radial direction and have also identified a longitudinal component of wall motion. It has also been observed that decreased longitudinal movement of the common carotid artery is associated with higher plaque burden^20^. Significantly lower amplitudes of both radial and longitudinal displacements have been found in older diabetic subjects, compared to healthy young adults^18^. Recently, the feasibility of assessing tissue motion inhomogeneities was demonstrated along with their association with the presence of coronary artery disease^21^. Blood pressure has been positively correlated with common carotid artery displacement^17^. Other studies have suggested that the severity of carotid stenosis is associated to axial wall stresses and accelerations^19^, as well as to the presence of an anterograde component in the longitudinal direction of wall motion^12^.

Related studies have proposed various metrics to quantify plaque motion patterns, including statistical measures of velocities, motion amplitudes and diastole-to-systole displacements of the entire plaque area during the cardiac cycle^11,13^, maximal (discrepant) surface velocities^9,22^ and displacement vector maps^23^. A group of studies have also qualitatively described the so called “jellyfish sign” phenomenon, according to which the carotid plaque surface rises and falls in a manner inconsistent with arterial pulsatile wall motion^24–26^. Other similar phenomena include motion of intraplaque contents^27^, mobility at the edge of the plaque, mobility in all parts of the plaque and mobility at the bottom of an ulcer on the plaque^26^. Studies have also investigated tissue strain, i.e. the change of displacement with respect to some initial reference status^10,28–32^. These studies have converged to the general conclusion that softer, echolucent plaques undergoing higher strains tend to be more prone to rupture and they are associated with poorer patient cognition. The concept of concordant and discordant motion was recently introduced to describe the spread of motion of different plaque areas^33^.

Among the investigated phenomena, relative motion between the plaque and the adjacent wall^13,24–26^, as well as within the plaque itself^26,27^ has been reported in some studies. The patterns of synchronisation of such relative movements have only been estimated qualitatively in a few studies^24–26^ and have shown that asynchronous motion of the plaque relative to the adjacent wall is associated with plaque instability and stroke recurrence.

To the best of our knowledge, there is no study focused on investigating synchronisation patterns of carotid plaque motion in an automated and quantitative way. Therefore, the purpose of this study was to quantify synchronisation patterns of the carotid plaque, in relation to its adjacent wall and within itself, and investigate potential associations of these synchronisation patterns with different plaque phenotypes, including echogenicity, stenosis degree, patient symptoms and plaque risk. The major contributions of this work are to (a) suggest a systematic approach for assessing such patterns, (b) provide specific numerical indices (measured in seconds) for the related phenomena, i.e. the phase shifts between plaque and wall, and within plaque in radial and longitudinal directions, and (c) evaluate the derived indices in different plaque phenotypes, based on the hypothesis that asynchronous plaque motion is associated with phenotypes characterising vulnerable plaque, namely echolucency, symptomaticity, high stenosis degree and high risk. These contributions will provide new knowledge about plaque biomechanics, which is important and necessary for future studies, including prognostic follow-up assessments.

## Materials and Methods

### Dataset

Seventy seven consecutive patients (59 men, 18 women) with carotid atherosclerosis were included in the study, free from comorbidities, including heart failure, liver dysfunction, cancer, chronic diseases etc. Subjects were on statin-based, anti-platelet and lipid-lowering medication. The dataset included 18 symptomatic patients (31 plaques, degrees of stenosis 66% ± 29%), 57 asymptomatic patients (98 plaques, degrees of stenosis 73% ± 22%) and 2 patients (6 plaques) whose symptomaticity or stenosis degree was unknown; the latter were only included in the association-with-echogenicity study. The symptomatic subjects, for whom only the ipsilateral artery was studied, had experienced a stroke or a TIA, within 6 months prior to the examination. A number of asymptomatic subjects had plaque in both the right and left carotids and in both types of subjects more than one plaque may be present in an artery (tandem lesions); tandem lesions were treated as separate plaques. The patients’ ages were 70 ± 9 years (range 43–85 years), and their stenosis degrees 75% ± 17% (range 20–99%), based on Doppler ultrasound measurements.

B- mode ultrasound images were acquired in longitudinal section using a LOGIQ Book (GE Medical Systems, Milwaukee, WI, USA) scanner and a linear array 4–10 MHz transducer. Subjects were examined in a supine position, with a slight backward inclination of the head, towards the opposite side of the carotid under examination. Patients rested for at least 5 minutes before the examination, to stabilise their heart rate and blood pressure. To minimise movements due to factors other than haemodynamic forces, the operator held the transducer as stable as possible, exerting minimal pressure, and the patients were asked to breath-hold during recordings. Scanner and transducer settings included a high dynamic range (60 or 75 dB) and zero persistence, and 10 MHz centre frequency. At least three cardiac cycles were recorded at a rate of 25 frames/s. Image resolution was 12 pixels/mm in the radial and longitudinal directions. The room temperature was kept constant at 26 °C.

All ultrasound examinations were performed by 4 experienced physicians in the Vascular Surgery Department of the University Hospital “ATTIKON”, Athens, Greece. Data collection was approved by the ATTIKON hospital institutional review board and all subjects included in the study gave their informed consent to the scientific use of the data. The methods were carried out in accordance with the relevant guidelines and regulations.

### Estimation of plaque motion synchronisation patterns

Plaque motion synchronisation patterns relative to the adjacent normal wall as well as within the plaque were estimated through cross-correlations of pairs of waveforms representing displacements of plaque and wall tissue.

#### Basic principles of cross-correlation

Cross-correlation ${r}_{d}$ is a measure of similarity of two signals in the form of time series, $x(i)$ and $y(i)$, where $i=1,2,\ldots ,{N}$ denotes time points, as a function of the displacement $d$ (also known as lag) of one relative to the other^34^. If cross-correlation is calculated for all lags $d=0,1,\ldots ,{N}-1$, then the resulting cross-correlation sequence is twice as long as that of the correlated series. The following formula for cross-correlation was used:

$${r}_{d}=\frac{\sum _{i}[(x(i)-{m}_{x})(y(i-d)-{m}_{y})]}{\sqrt{\sum _{i}{(x(i)-{m}_{x})}^{2}}\sqrt{\sum _{i}{(y(i)-{m}_{y})}^{2}}}$$

where ${m}_{x}$ and ${m}_{y}$ are the mean values of signals $x(i)$ and $y(i)$, respectively. The denominator in this formula serves to normalise the correlation coefficients, so that the cross-correlation is 1, for lag equal to 0. The subtraction of the mean values ${m}_{x}$ and ${m}_{y}$ from the signals allows signals from different subjects to be comparable. The length $N$ of the signals coincides with the maximum duration of the ultrasound recording in each case.

If the peaks (or the troughs) of two time-varying signals coincide in time, their cross-correlation has a high positive value. These signals are considered synchronous, or in-phase, or with a 0° phase shift. If the peaks of one signal coincide in time with the troughs of the other signal, their cross-correlation has a high negative value. These signals are considered asynchronous, or out-of-phase, or with a 180° phase shift. A cross-correlation value equal to 0 indicates uncorrelated signals.

#### Description of methodology

**Figure 1:**
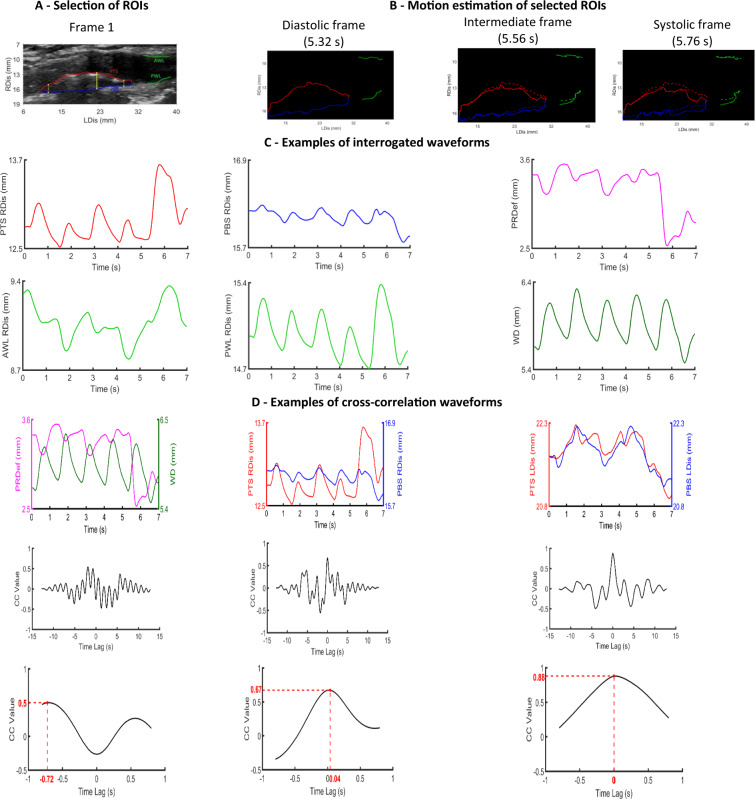
Examples of interrogated ROIs and corresponding waveforms, illustrating the different steps of the methodology. **A** – Plaque and wall ROIs in frame 1. Vertical yellow dotted lines indicate the boundaries of the investigated area. The top and bottom edges of the vertical yellow solid line correspond to the ROI pair for which waveforms are illustrated. **B** – Plaque and wall ROI pixel positions at three different frames representing different phases of the cardiac cycle, and corresponding time points, obtained after motion analysis. Pixel positions at diastole are superimposed on intermediate and systolic frames as dashed lines. **C** – Radial displacements of selected pixels of PTS, PBS and their difference (left column), and radial displacements of selected pixels of AWL, PWL and their difference, which represents the arterial wall diameter (right column). **D** – Displacement pairs for estimation of cross-correlation (top row), the corresponding cross-correlation waveforms (middle row) and the selected cross-correlation segment for calculation of features (bottom row). RDis: radial displacement, PRDef: plaque radial deformation, WD: wall diameter, LDis: longitudinal displacement.

The main steps of the methodology are described below and illustrated in Fig. 1.

##### Selection of regions of interest (ROIs)

For each plaque image sequence (video), an experienced physician marked manually in the first frame the following four ROIs: the posterior and anterior wall-lumen interfaces (PWL and AWL, respectively), and the plaque top and bottom surfaces (PTS and PBS, respectively) (Fig. 1A). PWL and AWL were selected on the normal, i.e. non-atheromatous, arterial wall, adjacent to the plaque.

##### Motion estimation of selected ROIs

The radial and longitudinal positions of all pixels included in the selected ROIs were estimated across all frames with an adaptive block-matching algorithm, which incorporates Kalman filtering^35^. This algorithm was evaluated in an *in silico* framework consisting of 13 simulated sequences, and has been shown to be accurate and robust in motion tracking of the arterial wall from B-mode ultrasound images^13^. For each ROI, 1.6 **×** 1 mm^2^ reference blocks were selected in the first frame, centred at ROI pixels. Figure 1B shows examples of selected ROIs (AWL, PWL, PTS, PBS) for a diastolic, an intermediate and a systolic frame of the sequence.

##### Waveforms extracted from motion analysis

ROI positions were used to estimate six sets of waveforms for each plaque:  

(i) wall diameter, which was selected as the most representative waveform, i.e. the one in which the most clear cyclic motion was observed, among the distances of vertical pairs of AWL and PWL pixels,
(ii) radial displacements of all PTS pixels, namely their radial positions along consecutive frames,
(iii) longitudinal displacements of all PTS pixels, namely their longitudinal positions along consecutive frames,
(iv) radial displacements of all PBS pixels,
(v) longitudinal displacements of all PBS pixels, and
(vi) radial distances of PTS and PBS pixel pairs, defined as the absolute differences of waveforms (ii), (iv) across vertical pixel pairs.

Twenty five pixels from the right and 25 from the left edge of the plaque PTS and PBS were removed to ensure that only plaque pixels, and no normal (non-plaque) wall area, were included in the analysis. The number of removed pixels (25) was heuristically determined, following visual inspection and testing. Figure 1C shows examples of interrogated waveforms.

A high-pass 4^th^ order Butterworth filter with a cutoff frequency of 0.6 Hz was applied to the displacement waveforms^36^, so as to remove unwanted offsets or abrupt fluctuations present in the low-frequency band. The cutoff value was selected to ensure that heart rates above approximately 40 beats per minute remain unaffected after filtering. Independent component analysis (ICA) demonstrated that the suggested methodology is robust against external motion (Supplementary methods).

##### Calculation of cross-correlations

Three types of cross-correlations were calculated using the previously described waveforms:

- Cross-correlation 1 (CC1): Radial deformation of the plaque with wall diameter, i.e. waveforms (i) and (vi),
- Cross-correlation 2 (CC2): Radial displacements of plaque top and bottom surfaces, i.e. waveforms (ii) and (iv), and
- Cross-correlation 3 (CC3): Longitudinal displacements of plaque top and bottom surfaces, i.e. waveforms (iii) and (v).

CC2 and CC3 describe intra-plaque kinematics, whereas CC1 was considered, so as to provide a measure with respect to a well-known arterial parameter.

Figure 1D shows examples of interrogated pairs of waveforms ((a)-(c), above) and their corresponding cross-correlations.

Signals to be correlated were confined within an average cycle window, estimated from the dominant frequency of the wall diameter waveform.

From each cross-correlation waveform, two types of measurements were obtained: (a) the sign corresponding to the maximum absolute cross-correlation, and (b) the corresponding lag *d*_*max*_, in seconds (Fig. 1D). For each plaque, cross-correlation waveforms were produced for all PTS-PBS pairs, and the following indices were then extracted:

- The synchronisation percentage, defined as the percentage of the positive values present in the entire set of maximum signed cross-correlation values, derived from all PTS-PBS pairs of the plaque. According to the principles of cross-correlation described previously, this percentage represents the proportion of plaque pairs that exhibit synchronous motion patterns for a given type of cross-correlation.
- Seven statistical (histogram-based) measures (maximum-, minimum-, mean-, median-value, standard deviation, skewness, and kurtosis) of the lags *d*_*max*_ extracted from all PTS-PBS pairs of the plaque.

Therefore a total of 24 features were extracted for each plaque, namely 8 features (synchronisation percentage and 7 statistical indices) for each of the 3 cross-correlation types.

### Grayscale normalisation and estimation of plaque echogenicity

To normalise ultrasound images according to widely accepted procedures^37^, the physician selected a region in the blood and one in the adventitia, and the median pixel values of these regions (GSM_blood_ and GSM_adv_, respectively) were set as the lowest (black) and the highest (white) values in the image, respectively. Then, the image grayscale intensities were linearly adjusted so that GSM_blood_ was 0, and GSM_adv_ was 190^37^.

An echolucent plaque is a dark appearing plaque in the ultrasound recording, while an echogenic plaque is a bright appearing one^38^. Plaque echogenicity was estimated as follows: the plaque was located automatically in each frame of the sequence after the first frame, using motion analysis of PBS and PTS areas, and the corresponding grayscale median (GSM) values were calculated. Plaque GSM was defined as the mean value of the GSMs of all frames. Echolucent plaques were considered those with a GSM < 25^39^ and echogenic those with GSM ≥ 25.

### Variability study

Intra and inter-observer variability were assessed by means of phase shift measurements performed for plaque boundaries displaced by 0–2 pixels with respect to the original (expert-annotated) ones. This experiment was designed based on the assumption that different observers, or the same observer at different times, produce different tissue outlines, which are displaced versions of a given contour. The range of the displacements (0–2 pixels, including subpixel values) was selected heuristically, based on observations that tissue outlines derived by different experts were not more than 2 pixels apart. Differences between original and displaced versions in all cases were assessed statistically.

### Classification & statistical analysis

The four associations investigated were validated through classification schemes using supervised machine learning. The purpose of classification was to evaluate the overall potential of the extracted features, which, can alternatively be considered as a “motion synchronisation signature”, through their association with the four clinical phenotypes. Subsequently, statistical analysis was performed, to identify the features with the highest discriminatory ability.

Feature selection was applied using Principal Component Analysis (PCA), whereby the initial feature set is converted into a reduced set of linearly uncorrelated features, orthogonal to each other (principal components), which retains most of the initial set’s variance, namely, its information content^40^. For this study, as many principal components as necessary were retained to cover 95% of the initial set’s variance.

Classification models for each association were implemented using the Weka workbench version 3.6 (Machine Learning Group at the University of Waikato, Hamilton, New Zealand)^41^. Among the algorithms available in Weka, the Random Forest (RF) algorithm was used, due to its superior performance and its robustness to overfitting^42^. The RF algorithm uses a number of parameters that need to be tuned properly, before training, to avoid overfitting or underfitting. The two parameters that were tuned included the number of features to be used in random selection (range: 2-number of features, with a step of 1), and the number of trees to be generated (range: 100–900, with a step of 200). For parameter tuning, 10-fold cross-validation was used. The parameters that were tuned included the number of data points, the number of features of each tree of the forest, and the number of the trees that we build for the forest.

To address the problem of class imbalance that is present in our data, the ADASYN algorithm^43^ was applied to create synthetic samples for the minority class, i.e. the class with the lowest number of cases. Of note, these synthetic samples were used only for training the model, not for testing.

For the evaluation of each model, leave-one-out cross-validation (LOOCV) was chosen, because the medium size of our dataset indicated it as the optimal choice in terms of computational cost, as well as bias-variance trade-off^44^.

To evaluate the performance of the classification models, a set of metrics was calculated, including accuracy (ACC), sensitivity (SENS), specificity (SPEC), precision (PREC), negative predictive value (NPV), F1 score (F1SC) and the area under the Receiver Operating Characteristics (ROC) curve (AUC)^45^.

Statistical analysis was performed using the non-parametric Wilcoxon rank sum test and statistical significance was considered for a p-value equal to or lower than 0.05.

All analyses were performed using Matlab R2016a (MathWorks, Natick, MA, USA) and a computer with an Intel Core i5 220 GHz CPU.

## Results

Table 1 shows the performance of the RF classifier, for the four associations interrogated, in terms of the evaluation metrics described in the previous section. This corresponds to the overall performance of all interrogated PCA-selected features.

**Table 1:** Values of evaluation metrics for the four associations investigated, corresponding to the overall performance of all interrogated PCA-selected features.

	ACC	SENS	SPEC	PREC	NPV	F1SC	AUC
*Echogenicity*	0.73	0.73	0.73	0.88	0.51	0.80	0.81
*Symptomaticity*	0.69	0.69	0.68	0.88	0.41	0.77	0.79
*Stenosis degree*	0.85	0.86	0.81	0.92	0.68	0.89	0.89
*Plaque risk*	0.84	0.83	0.88	0.96	0.58	0.89	0.90

Regarding the variability study, all indices were similar between the original and the displaced versions. As an example, the p-values for the mean phase shifts were 0.46 for CC1 and CC2 and 0.39 for CC3.

In the following subsections detailed results are presented for the statistical analysis of the entire dataset, for each of the investigated scenarios. Tables showing statistical analysis results present values for synchronisation percentages and mean phase shifts, even if they were not found statistically different, so as to provide a feel for these measures, given they are reported for the first time.

### Association with plaque echogenicity

Of the 135 plaques of the dataset, 37 were echolucent (GSM < 25) and 98 were echogenic (GSM ≥ 25). The stenosis degrees and ages were not statistically different in the two groups (p-values = 0.17 and 0.24, respectively).

The application of PCA identified 13 features as the principal components satisfying the 95% variance coverage criterion for this association.

Table 2 shows the mean values and corresponding p-values of the synchronisation percentages, mean phase shift values, and statistically significant features for the three cross-correlation types, in echogenic and echolucent plaques. As we can see, in echolucent plaques, the top plaque surface moves less synchronously (with a higher phase shift) relative to the bottom surface, than in echogenic plaques, in the radial direction (higher mean_CC2_ and median_CC2_). Also, the mean phase shifts between top and bottom surfaces of both echogenic and echolucent plaques were significantly higher in the longitudinal direction, compared to the radial direction.

**Table 2:** Mean ± standard deviation values and corresponding p-values of the synchronisation percentages, mean phase shift values, and statistically significant features for the three cross-correlation types, for echogenic and echolucent plaques.

	Echogenic	Echolucent	p-value
sp_CC1_	52% ± 24%	60% ± 23%	0.11
sp_CC2_	82% ± 17%	81% ± 15%	0.47
sp_CC3_	77% ± 18%	74% ± 16%	0.22
mean_CC1_ (s)	0.42 ± 0.20	0.40 ± 0.19	0.83
mean_CC2_ (s)	0.20 ± 0.15	0.26 ± 0.15	**0.05**
mean_CC3_ (s)	0.30 ± 0.18*	0.34 ± 0.15*	0.09
median_CC2_ (s)	0.09 ± 0.17	0.11 ± 0.13	**0.05**

sp: synchronisation percentage

* indicates significant difference (p-value < 0.05) with respect to mean_CC2_.

### Association with symptomaticity

Of the 124 plaques used in this substudy, 93 caused a degree of stenosis higher than or equal to 70%. Of these 93 high-stenosis plaques, 71 were asymptomatic and 22 were symptomatic. The stenosis degrees and ages were not statistically different in the two groups (p-values = 0.15 and 0.35, respectively).

The application of PCA identified 11 features as the principal components satisfying the 95% variance coverage criterion for this association.

Table 3 shows the mean values and corresponding p-values of the synchronisation percentages, mean phase shift values, and statistically significant features for the three cross-correlation types, for asymptomatic and symptomatic plaques. As we can see, there was no difference between symptomatic and asymptomatic cases (except for 3 histogram-based features). Also, the mean phase shifts between top and bottom surfaces of asymptomatic plaques were significantly higher in the longitudinal direction, compared to the radial direction. Symptomatic plaques did not show such difference.

**Table 3:** Mean ± standard deviation values and corresponding p-values of the synchronisation percentages, mean phase shift values, and statistically significant features for the three cross-correlation types, for asymptomatic and symptomatic plaques of high stenosis degrees.

	Asymptomatic	Symptomatic	p-value
sp_CC1_	57% ± 23%	53% ± 21%	0.38
sp_CC2_	82% ± 15%	79% ± 14%	0.34
sp_CC3_	80% ± 14%	76% ± 19%	0.36
mean_CC1_ (s)	0.40 ± 0.20	0.47 ± 0.18	0.13
mean_CC2_ (s)	0.23 ± 0.14	0.24 ± 0.13	0.51
mean_CC3_ (s)	0.29 ± 0.15*	0.32 ± 0.18	0.64
max_CC1_ (s)	1.02 ± 0.20	1.14 ± 0.13	**0.01**
stdev_CC1_ (s)	0.33 ± 0.11	0.39 ± 0.09	**0.05**
max_CC3_ (s)	0.96 ± 0.27	1.06 ± 0.24	**0.05**

sp: synchronisation percentage, stdev: standard deviation.

*indicates significant difference (p-value < 0.05) with respect to mean_CC2_.

### Association with stenosis degree

Of the 124 plaques used in this substudy, 97 were asymptomatic. Of these 97 asymptomatic plaques, 26 caused a low degree of stenosis (<70%) and 71 caused a high degree of stenosis (≥70%). The ages of the patients were not statistically different in the two groups (p-value = 0.16). By definition, the high-stenosis group in this study is the same as the asymptomatic group in the previous study.

The application of PCA identified 13 features as the principal components satisfying the 95% variance coverage criterion, for this association.

Table 4 shows the mean values and corresponding p-values of the synchronisation percentages, mean phase shift values, and statistically significant features for the three cross-correlation types, for low- and high-stenosis plaques. As we can see, in high-stenosis plaques, the top plaque surface moves less synchronously (higher max_CC2_, higher mean_CC2_) and less uniformly (higher stdev_CC2_) relative to the bottom surface, than in low-stenosis plaques, in the radial direction. Also, the mean phase shifts between top and bottom surfaces of both low- and high-stenosis plaques were significantly higher in the longitudinal direction, compared to the radial direction.

**Table 4:** Mean ± standard deviation values and corresponding p-values of the synchronisation percentages, mean phase shift values, and statistically significant features for the three cross-correlation types, for low-stenosis and high-stenosis plaques.

	Low-stenosis	High-stenosis	p-value
sp_CC1_	50% ± 27%	57% ± 23%	0.27
sp_CC2_	85% ± 16%	82% ± 15%	0.14
sp_CC3_	75% ± 18%	80% ± 14%	0.23
mean_CC1_ (s)	0.41 ± 0.20	0.40 ± 0.20	0.98
mean_CC2_ (s)	0.16 ± 0.15	0.23 ± 0.14	**0.03**
mean_CC3_ (s)	0.27 ± 0.15*	0.29 ± 0.15*	0.49
min_CC1_ (s)	0.03 ± 0.07	0.02 ± 0.08	**0.03**
max_CC2_ (s)	0.66 ± 0.41	0.94 ± 0.26	**0.00**
stdev_CC2_ (s)	0.19 ± 0.13	0.29 ± 0.12	**0.00**

sp: synchronisation percentage, stdev: standard deviation.

*indicates significant difference (p-value < 0.05) with respect to mean_CC2_.

### Association with plaque risk

Of the 124 plaques used in this substudy, 26 were low-risk and 98 were high-risk. The ages of the patients were not statistically different in the two groups (p-value = 0.25). According to the current clinical decision-making scheme, high-risk subjects are symptomatic ones with stenosis degrees ≥50%^46^ and asymptomatic subjects with stenosis degrees ≥70%^47^; otherwise subjects are considered low-risk^5^.

The application of PCA identified 12 features as the principal components satisfying the 95% variance coverage criterion for this association.

Table 5 shows the mean values and corresponding p-values of the synchronisation percentages, mean phase shift values, and statistically significant features for the three cross-correlation types, for low- and high-risk plaques. As we can see, in high-risk plaques, the top plaque surface moves less synchronously (higher mean_CC2_) and less uniformly (higher stdev_CC2_) relative to the bottom surface, than in low-risk plaques, in the radial direction. In addition to this, most of the significantly different features (3 out of 4) were derived from cross-correlation type 2, namely between radial motion of top and bottom plaque surfaces. Also, the mean phase shifts between top and bottom surfaces of both low-risk and high-risk plaques were significantly higher in the longitudinal direction, compared to the radial direction.

**Table 5:** Mean ± standard deviation values and corresponding p-values of the synchronisation percentages, mean values and statistically significant features for the three cross-correlation types, for low-risk and high-risk plaques.

	Low-risk	High-risk	p-value
sp_CC1_	50% ± 27%	56% ± 22%	0.34
sp_CC2_	85% ± 16%	81% ± 15%	0.08
sp_CC3_	75% ± 18%	79% ± 16%	0.36
mean_CC1_ (s)	0.41 ± 0.20	0.41 ± 0.20	0.79
mean_CC2_ (s)	0.16 ± 0.15	0.23 ± 0.14	**0.02**
mean_CC3_ (s)	0.27 ± 0.15*	0.30 ± 0.16*	0.35
min_CC1_ (s)	0.03 ± 0.07	0.02 ± 0.07	**0.04**
max_CC2_ (s)	0.66 ± 0.41	0.94 ± 0.27	**0.00**
stdev_CC2_ (s)	0.19 ± 0.13	0.29 ± 0.12	**0.00**

sp: synchronisation percentage, stdev: standard deviation.

*indicates significant difference (p-value < 0.05) with respect to mean_CC2_.

As it can be observed, a few of the features in the previous Tables 2, 4 and 5 present high standard deviations, sometimes even higher than the corresponding mean values (median_CC2_ in Table 2, and min_CC1_ in Tables 4 and 5), indicating a high inter-plaque variability, probably due to differences between subjects.

### Representative examples of cross-correlation distributions

**Figure 2:**
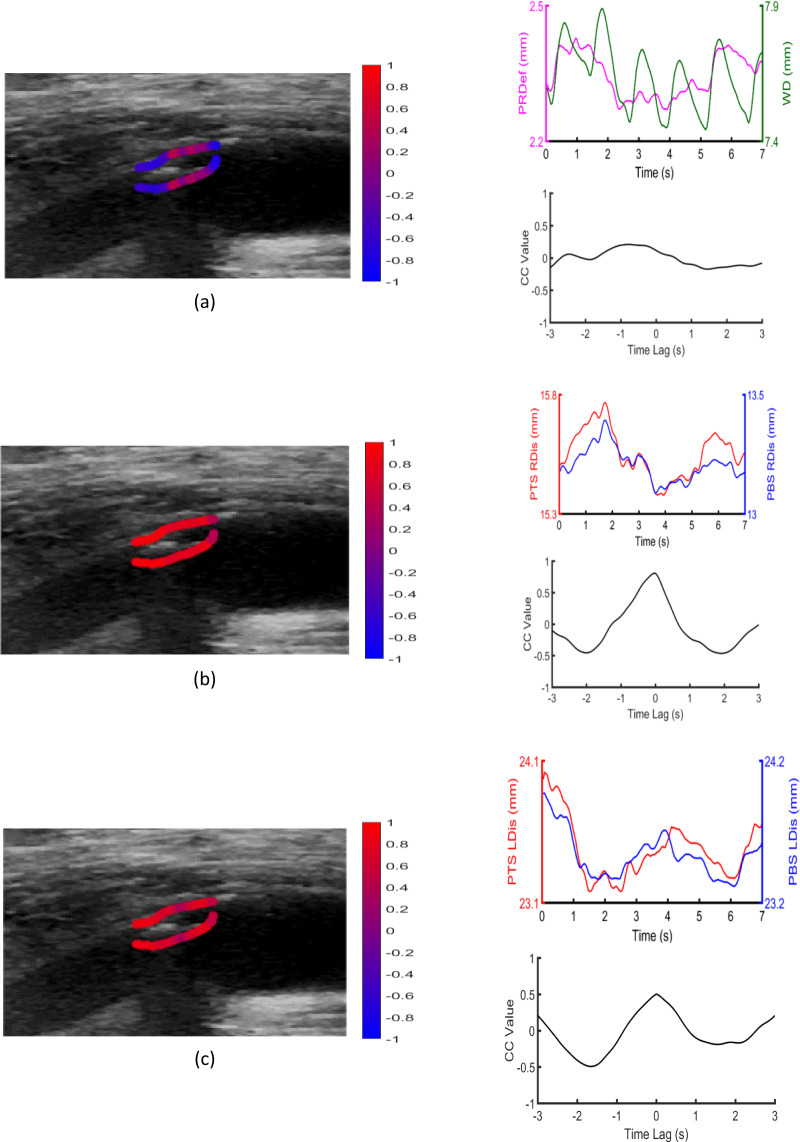
Example of an echogenic (GSM = 30) low-stenosis (60%) asymptomatic plaque, with (**a**) contours superimposed on the B-mode image, illustrating the distribution of CC1 values on PTS and PBS (top row), and displacement waveforms of the central pixel pair and the corresponding cross-correlation waveform (bottom row), (**b**) contours superimposed on the B-mode image, illustrating the distribution of CC2 values on PTS and PBS (top row), and displacement waveforms of the central pixel pair and the corresponding cross-correlation waveform (bottom row) and (**c**) contours superimposed on the B-mode image, illustrating the distribution of CC3 values on PTS and PBS (top row), and displacement waveforms of the central pixel pair and the corresponding cross-correlation waveform (bottom row). RDis: radial displacement, PRDef: plaque radial deformation, WD: wall diameter, LDis: longitudinal displacement.

**Figure 3:**
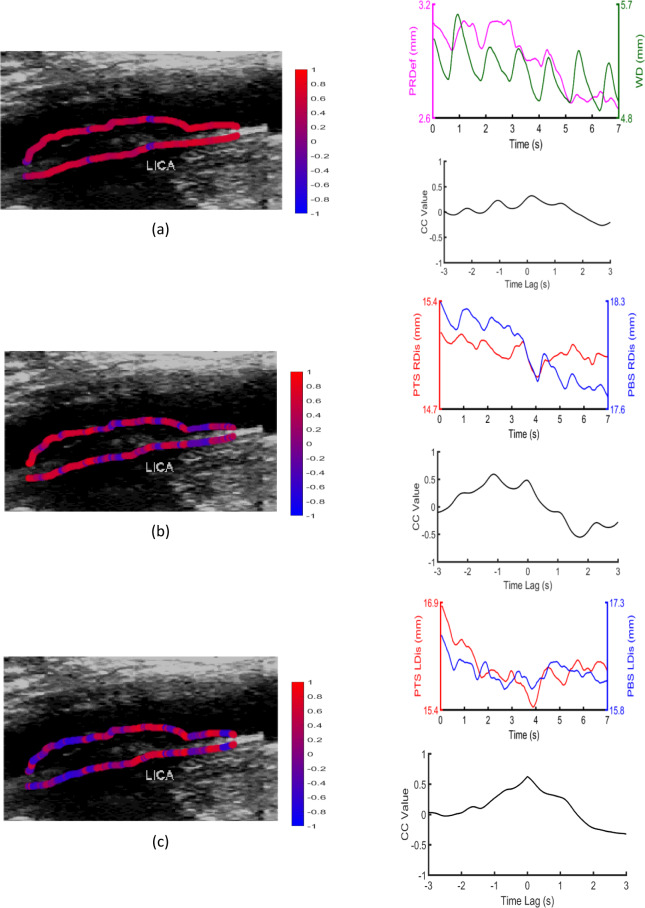
Examples of an echolucent (GSM = 15) high-stenosis (70%) symptomatic plaque, with (**a**) contours superimposed on the B-mode image, illustrating the distribution of CC1 values on PTS and PBS (top row), and displacement waveforms of the central pixel pair and the corresponding cross-correlation waveform (bottom row), (**b**) contours superimposed on the B-mode image, illustrating the distribution of CC2 values on PTS and PBS (top row), and displacement waveforms of the central pixel pair and the corresponding cross-correlation waveform (bottom row) and (**c**) contours superimposed on the B-mode image, illustrating the distribution of CC3 values on PTS and PBS (top row), and displacement waveforms of the central pixel pair and the corresponding cross-correlation waveform (bottom row). RDis: radial displacement, PRDef: plaque radial deformation, WD: wall diameter, LDis: longitudinal displacement.

Figures 2 and 3 illustrate examples of distributions of cross-correlations of the three types of cross-correlations for an echogenic, asymptomatic, low-stenosis case and an echolucent, symptomatic, high-stenosis case, respectively. Cross-correlation values correspond to pixels along the manually extracted plaque contour in the first frame of the sequence. Videos 1 and 2 show the displacements of the interrogated ROIs (AWL, PWL, PTS and PBS) in each case. Synchronisation percentages were 38%, 100% and 100% for CC1, CC2 and CC3, respectively, in the asymptomatic case and 95%, 72% and 53% for CC1, CC2 and CC3, respectively, in the symptomatic case. Mean phase shifts were 0.45 s, 0.00 s and 0.04 s for CC1, CC2 and CC3, respectively, in the asymptomatic case and 0.79 s, 0.33 s and 0.48 s for CC1, CC2 and CC3, respectively, in the symptomatic case.

## Discussion

This study showed that the synchronisation percentages in our dataset were approximately 50%, 80% and 80%, for CC1, CC2 and CC3, respectively, and the mean phase shifts were 0.4 s, 0.2 s and 0.3 s, respectively. To the best of our knowledge, such features characterising phase shifts and synchronisation percentages of the motion of carotid atheromatous plaque from B-mode ultrasound have not been previously quantified. The RF algorithm yielded AUC scores of 0.81, 0.79, 0.89 and 0.90, for the association with echogenicity, symptomaticity, stenosis degree and plaque risk, respectively. It was also observed that echolucent, high-stenosis and high-risk plaques had significantly higher phase shifts between the radial displacements of their top and bottom surfaces (0.23–0.26 s on average), compared to echogenic, low-stenosis and low-risk plaques (0.16–0.20 s on average).

The interrogated phenotypes were selected on the grounds of their associations with plaque vulnerability and selection of treatment. Specifically, echogenicity has been associated with increased vulnerability. Symptomatic and asymptomatic plaques with stenosis degrees higher than 70% are currently offered carotid revascularisation^5^. Asymptomatic subjects with low- and high-stenoses are offered different treatments; conservative treatment with medication for the former, while carotid revascularisation for the latter^5^.

Feature selection identified the same set of features for most association scenarios (3 out of 4, with a small differentiation for the symptomaticity scenario). Also, in all association studies, NPV had the lowest value among all evaluation metrics. This is expected, because the “negative” class was the minority class, namely it was outnumbered by the “positive” class, therefore, this metric reflects the inferiority of the “negative” class in terms of sample size. It is pointed out that 3 additional classifiers, besides RF, were benchmarked on the same dataset, namely Multilayer Perceptron, Nearest Neighbours and Support Vector Machines (SVMs). These algorithms perform supervised machine learning, i.e. their inputs and outputs are known; see^48^ for more information. The performances of these classifiers were inferior compared to the RF algorithm.

Although feature selection identified 12–13 features for each association scenario, statistical analysis yielded fewer features, namely 2–4 depending on the scenario. This indicates that despite the relatively low number of statistically significant features in a specific association, there is additional, potentially discriminatory, information which is uniformly distributed among the entire set of the 24 features, and is revealed with classification. The good performance of the classifiers, ranging from 79% to 90%, indicates that there is sufficient information present in the datasets of all association scenarios.

Most of the previous studies have used statistical tests to validate their results^9,13,22,25–27^, while machine learning methodologies have been introduced in fewer cases^11,30,32,24^. Specifically, Gastounioti *et al*.^11^ compared multiple classifiers and feature selection methods, as well as combinations of them, and concluded that the SVM classifier combined with the Fisher Discriminant Ratio for feature selection were optimal in discriminating symptomatic and asymptomatic patients. Meshram *et al*.^30^ and Wang *et al*.^32^ implemented a logistic regression classifier and ROC analysis, towards correlation of plaque strain indices with patient cognitive function. Finally, Ichinose *et al*.^24^ implemented a multiple linear regression analysis (stepwise analysis and partial least squares analysis), followed by a machine learning analysis using an Artificial Neural Network based on the Log-Linearised Gaussian Mixture Network, to correlate the “jellyfish sign” of motion with the presence of new lesions, detected by diffusion-weighted imaging. The generation of these lesions is the most common complication caused by carotid artery stenting. Machine learning is appropriate for the study of complex relations, whereas statistical tests are limited to simpler cases. The combination of both machine learning and statistical analysis methodologies, which is implemented in the current study, allows the design of a robust, multi-level validation scheme and, thus, the extraction of reliable results about the complex phenomenon of plaque motion synchronisation.

Echolucent, high-stenosis and high-risk plaques presented significantly higher phase shifts between the radial displacements of their top and bottom surfaces, compared to echogenic, low-stenosis and low-risk plaques. A potential implication of these findings is that asynchronous motion patterns are associated with higher plaque vulnerability, given their association with its determinants, including echolucency, high-stenosis and presumed high risk. These results and related implications should be confirmed in follow-up studies. In contrast, statistical analysis between symptomatic and asymptomatic plaques did not reveal any differences. This finding may imply that echogenicity and stenosis degree hold more information and, thus, are more crucial clinical parameters, than symptomaticity, as far as plaque kinematics are concerned. Moreover, the significantly higher phase shifts in the longitudinal direction, in the majority of interrogated groups (7 out of 8), indicate more asynchronous intra-plaque motion in the longitudinal direction, than in the radial direction.

The main findings of this research, namely that echolucent, high-stenosis and high-risk plaques are characterised by higher phase shifts and, thus, less synchronous motion patterns between the radial motion of their top and bottom surfaces than echogenic, low-stenosis and low-risk plaques, qualitatively agree with other studies on plaque kinematics. Gastounioti *et al*.^13^ reported that symptomatic plaques presented 37% higher radial motion range of PTS and 50% higher relative movement between PTS and PBS. Moreover, Kume *et al*.^25^, Ogata *et al*.^26^ and Ichinose *et al*.^24^ showed that the jellyfish sign, a pattern that characterises the asynchronous motion of the plaque relative to the adjacent wall, is associated with plaque vulnerability and stroke recurrence. Gastounioti *et al*.^49^ found that echolucent plaque segments moved more intensely in the radial direction, compared to echogenic plaque segments. Finally, Tat *et al*.^12^ reported that patients with severe plaque stenosis presented greater longitudinal anterograde wall motion than those with moderate stenosis. In combination with our finding that high-stenosis plaques had significantly higher and more dispersed phase shifts between the radial displacement of their top and bottom surfaces, this suggests that irregular wall dynamics characterising high-stenosis cases may be reflected not only within plaque but also in relative movement with the adjacent wall.

This work is one of the studies demonstrating the ability to extract features characterising tissue kinematics from B-mode ultrasound images. Although radiofrequency ultrasound is being widely used for tissue motion and strain estimation^23,31,32^, B-mode has also been used for motion measurements^22,24,26,29^. In this work, only B-mode data were available in the commercial scanning device that was used. It has been shown that radiofrequency ultrasound outperforms B-mode, due to its reduced variability in cardiac strain estimation^50^. A more recent study however showed that local arterial characteristics can be assessed equally reliably and accurately with B-mode technology^51^. Advantages of B-mode include relatively low-cost and widespread use in clinical practice, while radiofrequency devices are higher-cost and mostly used for research purposes. It is therefore important to be able to extract as much information as possible from the widely available B-mode devices allowing to address a wider range of clinical applications. B-mode-ultrasound-based tissue kinematics could be further combined with other plaque properties, such as neovascularisation and elasticity, assessed using contrast-enhanced ultrasound and elastography, respectively, towards providing an overall valid plaque characterisation^52^.

Motion of the arterial wall and plaque during the cardiac cycle is a particularly complex phenomenon, resulting from the combined effect of a number of different forces/stresses, including translation, rotation, shear, tethering, etc. Taking into account the complexity of this phenomenon, in this study we selected to address representative plaque motion patterns, namely in relation to adjacent wall as well as in the radial and longitudinal directions within itself.

The limitations of this study include the medium size and the heterogeneity of the dataset. Compared to previous studies on ultrasound-based carotid plaque kinematics, in which dataset sizes ranged from 11 to 165 patients, our 77-patient (135-plaque) dataset was considered adequate for benchmarking our methodology. Dataset heterogeneity consists in including subjects of both gender and with lesions located in both the left and right carotids. Although larger and more homogeneous datasets are always desirable to reach safer conclusions, we believe that the medium size of our dataset and the grouping into smaller, somewhat more homogeneous, datasets has allowed us to make some reliable and interesting observations.

The findings presented in this study are promising for further in-depth study of carotid plaque kinematics from B-mode ultrasound. Future work in this area might focus on the combination of phase-shift features with other ultrasound-based kinematic features towards extracting valuable information about plaque mechanics. The application of the proposed classification model to substantially larger datasets, including follow-up patient data, will allow the identification of potential novel markers for improved risk stratification.

In conclusion, this study quantified synchronisation patterns of the carotid atheromatous plaque from B-mode ultrasound, and associated them with echogenicity, symptomaticity, stenosis degree and plaque risk. Synchronisation percentages in our dataset were approximately 50%, 80% and 80% and the mean phase shifts 0.4 s, 0.2 s and 0.3 s, for cross-correlation types 1, 2 and 3, respectively. The RF algorithm, combined with PCA, achieved very good performance in the benchmarking procedures, yielding AUC scores of 0.81, 0.79, 0.89 and 0.90, for the association with echogenicity, symptomaticity, stenosis degree and plaque risk, respectively. Statistical analysis showed that echolucent, high-stenosis and high-risk plaques exhibited higher phase shifts between the radial displacements of their top and bottom surfaces. These findings are promising for further in-depth study of ultrasound-based carotid plaque kinematics, towards improving risk stratification.

## Supplementary information

Video 1.

Video 2.

Independent Component Analysis.

## Data Availability

The datasets generated and analysed during the current study are available from the corresponding author on reasonable request.
