# Supplementary material for: Motion synchronisation patterns of the carotid atheromatous plaque from B-mode ultrasound
Source: Sci Rep. 2020 Jul 8;10:11221. doi: 10.1038/s41598-020-65340-2 (PMC7343786; doi:10.1038/s41598-020-65340-2)
Supplement: Supplementary file 3 — Independent Component Analysis. [file 41598_2020_65340_MOESM3_ESM.docx]

# **Motion synchronisation patterns of the carotid atheromatous plaque from B-mode ultrasound**

Spyretta Golemati, Eleni Patelaki, Aimilia Gastounioti, Ioannis Andreadis, Christos D. Liapis, Konstantina S. Nikita

Supplementary Methods

**Independent Component Analysis**

For each plaque, Independent Component Analysis (ICA) was performed for each set of vertical PBS-PTS pixel pairs (2 waveforms – radial/longitudinal displacement of each point of the pair). So, after obtaining two independent components from the algorithm, we computed the cross-correlation between the independent component waveforms and the original plaque point waveforms.

The results of this analysis showed that, for over 80% of all vertical pairs, on average among all plaques, the two independent components correlated well (correlation value ≥ 0.65) with the two original waveforms, in such a way that one independent component correlated well with one and only one original plaque waveform, and vice versa. Specifically, the percentages of the pairs for which this pattern was observed were 85% ± 14% for radial direction and 82% ± 14% for longitudinal direction. Therefore, the original waveforms are maintained well after the application of ICA. A reasonable interpretation of this observation is that, in the majority of cases, the noise introduced by potential unwanted motion is not significant enough to affect our analysis and its results.

| 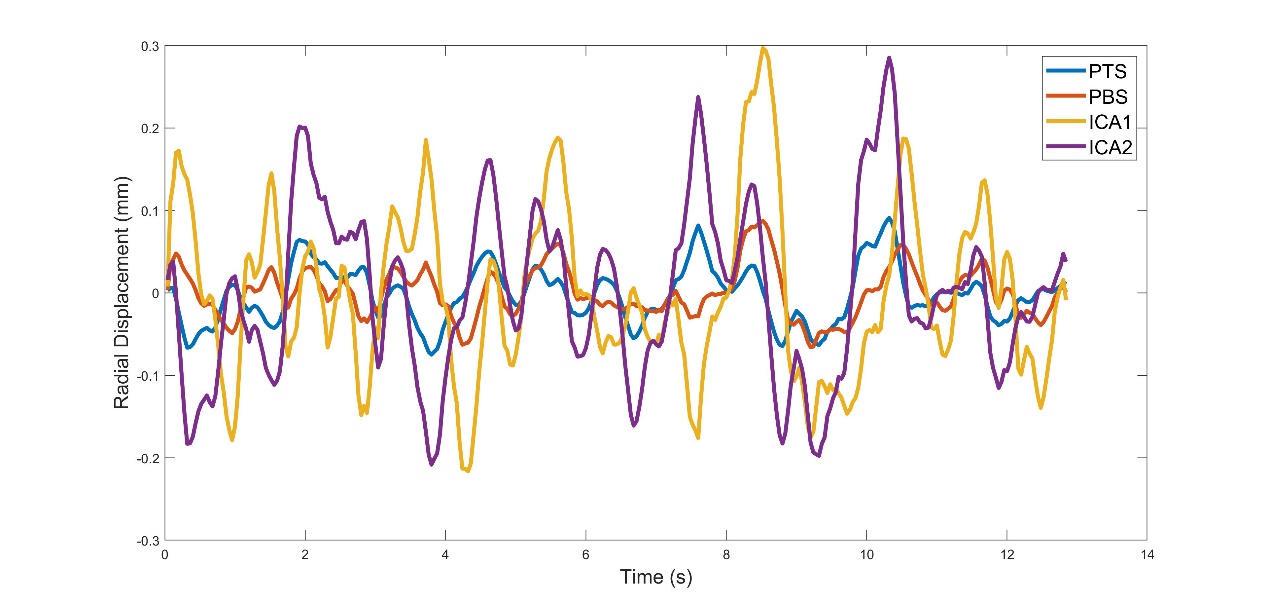  (a) |
| --- |
| 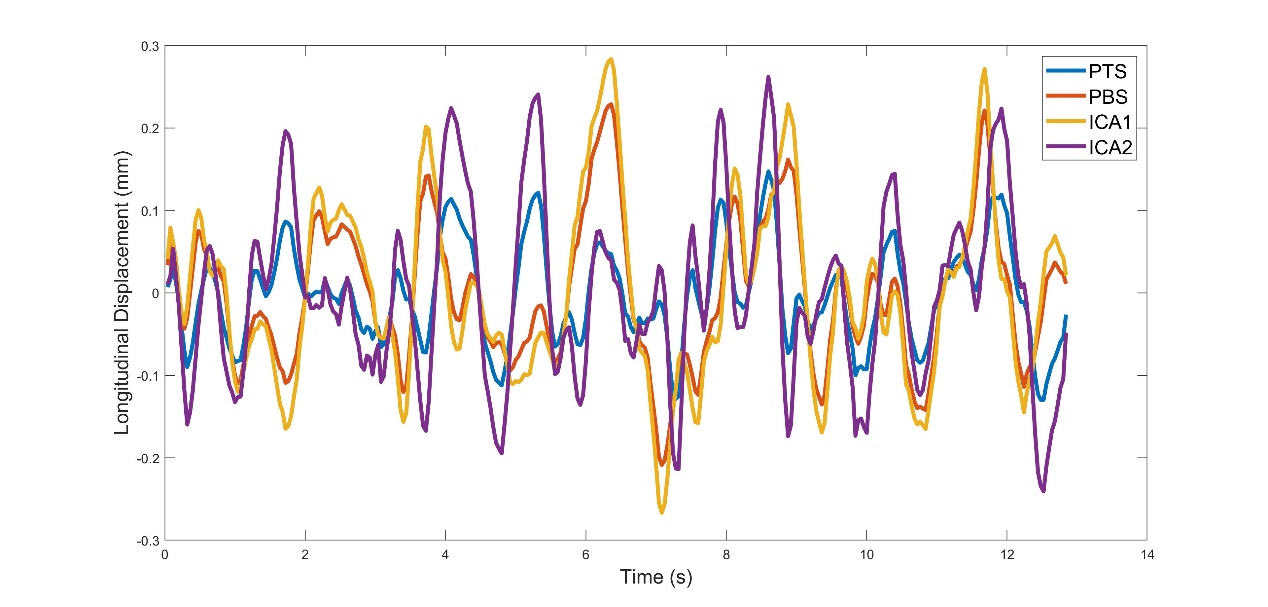  (b) |
| Figure S1. (a) correlation of ICA1 with PBS (yellow with orange) = 0.94; correlation of ICA2 with PTS (purple with blue) = 0.99. (b) correlation of ICA1 with PBS (yellow with orange) = 0.99; correlation of ICA2 with PTS (purple with blue) = 0.98 . |
